# Isolation, marine transgression and translocation of the bare‐nosed wombat (*Vombatus ursinus*)

**DOI:** 10.1111/eva.12785

**Published:** 2019-03-21

**Authors:** Alynn Martin, Scott Carver, Kirstin Proft, Tamieka A. Fraser, Adam Polkinghorne, Sam Banks, Christopher P. Burridge

**Affiliations:** ^1^ School of Natural Sciences University of Tasmania Hobart Tasmania Australia; ^2^ Animal Research Centre University of the Sunshine Coast Sippy Downs Queensland Australia; ^3^ College of Engineering, IT and Environment Charles Darwin University Casuarina Northern Territory Australia

**Keywords:** conservation, genetic structure, island biogeography, population genetics, spatial structure, *Vombatus ursinus*

## Abstract

Island populations can represent genetically distinct and evolutionarily important lineages relative to mainland conspecifics. However, phenotypic divergence of island populations does not necessarily reflect genetic divergence, particularly for lineages inhabiting islands periodically connected during Pleistocene low sea stands. Marine barriers may also not be solely responsible for any divergence that is observed. Here, we investigated genetic divergence among and within the three phenotypically distinct subspecies of bare‐nosed wombats (*Vombatus ursinus*) in south‐east Australia that are presently—but were not historically—isolated by marine barriers. Using genome‐wide single nucleotide polymorphisms, we identified three genetically distinct groups (mainland Australia, Bass Strait island, and Tasmania) corresponding to the recognized subspecies. However, isolation by distance was observed in the Tasmanian population, indicating additional constraints on gene flow can contribute to divergence in the absence of marine barriers, and may also explain genetic structuring among fragmented mainland populations. We additionally confirm origins and quantify the genetic divergence of an island population 46 years after the introduction of 21 individuals from the Vulnerable Bass Strait subspecies. In the light of our findings, we make recommendations for the maintenance of genetic variation and fitness across the species range.

## 1 INTRODUCTION

Islands are frequently the location of populations that can be phenotypically distinguished from those elsewhere (e.g., Harmon & Gibson, 2006; Schlotfeldt & Kleindorfer, 2006), and contribute to global biodiversity through the effects of isolation on genetic divergence and speciation (Wilson et al., 2008). Islands also represent important reservoirs for biodiversity, often removed from threats experienced on other landmasses, such as introduced pests (Short, Kinnear, & Robley, 2002). However, island populations can also be of elevated conservation concern, given lower abundances, lack of connectivity, lower genetic diversity and susceptibility to genetic drift (Frankham, 1997). Continental shelf islands are distinctive in this context, experiencing periods of connection to larger landmasses via land bridges during glacial periods when sea levels are low (most recently in the Pleistocene; Burridge, 2012). Depending upon the timing, duration and frequency of these connections, and the nature of intervening habitats, gene flow may have been experienced between lineages occupying presently isolated regions. This raises questions regarding their conservation prioritization given uncertainty about their history of genetic isolation. Furthermore, phenotypic distinction of lineages on continental shelf islands may also be problematic to interpret if the peripheral geographic setting of these islands confers environmental differences (Mullen, Vignieri, Gore, & Hoekstra, 2009), in addition to potential influences of island size alone (e.g., dwarfism in island emus; Thomson et al., 2018). This is a question of broad conservation interest, as continental shelf islands are common and host high biodiversity, most notably in South‐East Asia (e.g., the entire Malay Archipelago), but also Europe (e.g., England and many islands of the Mediterranean), North America (e.g., Newfoundland), South America (e.g., Falkland Islands) and Australia (e.g., Tasmania; Burridge, 2012).

Historical sea‐level rise associated with the end of the last glacial maximum (LGM) potentially played a significant role in the biogeography of south‐eastern Australia. This event isolated Tasmania and an array of islands from continental Australia during the flooding of Bass Strait, protecting some populations from causes of extinction that are present on the mainland (e.g., invasive predators; Kinnear, Sumner, & Onus, 2002), and shaping the population genetic structure of others (Firestone, Elphinstone, Sherwin, & Houlden, 1999; Toon, Mather, Baker, Durrant, & Hughes, 2007). These areas were connected by the Bassian land bridge during the LGM circa 25 kya (Lambeck & Chappell, 2001). As sea level rose, the mainland, Tasmania, and intervening islands remained connected through a western sill until around 17.5 kya and an eastern sill until around 14 kya (Lambeck & Chappell, 2001). Many species still occur across these now isolated regions, with Bass Strait and offshore Tasmanian islands exhibiting high species richness per unit area relative to other Australian islands (Burbidge, Williams, & Abbott, 1997), and supporting populations of mammals which are now extinct or declining on mainland Australia (Morris et al., 2018). These island populations may represent important genetic lineages and evolutionary legacies that are distinct from the mainland (e.g., platypus; Furlan et al., 2012), or may be representative of the mainland genetic pool (e.g., white‐bellied sea‐eagles; Shephard, Hughes, Catterll, & Olsen, 2005).

**Figure 1:**
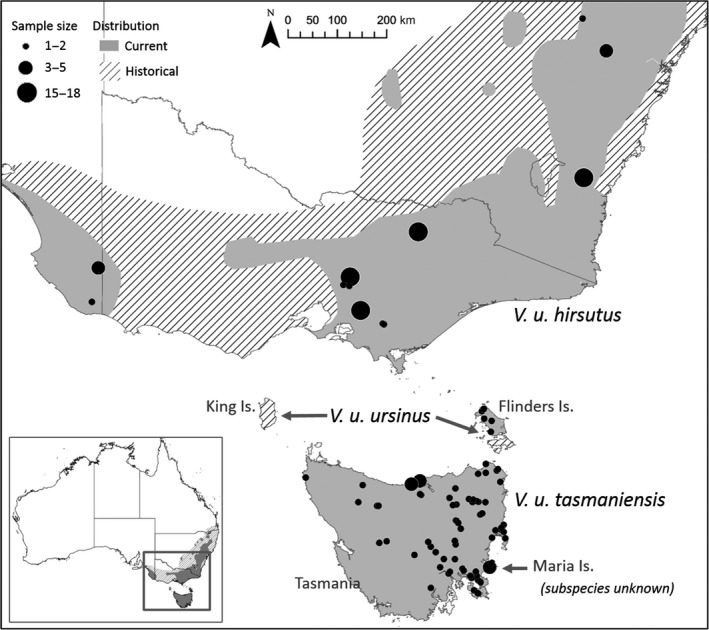
The bare‐nosed wombat distribution across Australia. Sampling locations and sample size are indicated by the circles (Supporting information Data S1 for location coordinates). Spatial data for the current distribution accessed from the International Union for Conservation of Nature (IUCN, 2016)

Wombats are evolutionarily significant as the largest extant burrowing mammals (Johnson, 1998). The bare‐nosed wombat (*Vombatus ursinus*) is a large (up to 50 kg), fossorial marsupial endemic and historically widespread in south‐east Australia (mainland and islands, Figure 1; Triggs, 2009; IUCN, 2016). Within this range, there are three recognized allopatric subspecies: south‐eastern mainland (*Vombatus u. hirsutus*; Perry 1810), Bass Strait islands (*V. u. ursinus*; Shaw 1800) and Tasmanian (*Vombatus u. tasmaniensis*; Spender and Kershaw, 1910) (Jackson, 2015). These subspecies are distinguished based on distribution and body size, with mainland individuals being the largest and Flinders Island being the smallest (Tate, 1951)—though these distinctions are in need of revisitation in an updated and comprehensive way. Despite being considered “common”—*V. ursinus* Least Concern on IUCN Red List (Taggart, Martin, & Menkhorst, 2016)—all three subspecies have experienced range retractions since settlement by Europeans (Figure 1), and may support several genetically important, yet isolated populations. Specifically, the range of *V. u. hirsutus* has been fragmented and more than halved, and similar retraction has been observed in *V. u. ursinus*, which now exists only on Flinders Island, having gone extinct on King, Cape Barren, Deal and Clarke islands (Rounsevell, Taylor, & Hocking, 1991). The Tasmanian subspecies exists throughout Tasmania with seemingly stable populations across its range (Figure 1; DPIPWE, 2017). A growing population also exists on Maria Island (Figure 1; Ingram, 2015), which may represent the descendants of 21 individuals translocated from Flinders Island (Rounsevell, 1989), and hence potentially of conservation significance for *V. u. ursinus*. However, records are inconsistent as to whether *V. ursinus* existed on Maria Island prior to this translocation event (Plomley, Cornell, & Banks, 1990; Rounsevell et al., 1991).

Despite range retractions observed in *V. ursinus*, it is still distributed relatively continuously, but with areas of fragmentation in the western and northern edges of the mainland distribution (IUCN, 2016). Assessing genetic structure within subspecies could reveal important biological processes, such as dispersal limitations and barriers to gene flow, that are also relevant for conservation with respect to the maintenance of genetic diversity. Evidence for isolation by distance has been observed for *V. u. hirsutus*, with high levels of population differentiation at larger spatial scales (Banks, Skerratt, & Taylor, 2002). However, sampling in this study was spatially clumped, and patterns of genetic structure and isolation by distance should be addressed within a continuously sampled region (Bradburd, Coop, & Ralph, 2018; Rosenberg et al., 2005). Assessing genetic structure within regions (mainland and Tasmania) also provides a valuable contrast for genetic structuring that may be ascribed to isolation by historical sea‐level rise.

Here, we utilize genome‐wide single nucleotide polymorphisms (SNPs) to (a) quantify the population structure of bare‐nosed wombats across their current range in the context of the presently recognized subspecies and their potentially dynamic history of connectivity, (b) document within region genetic variation to assess gene flow within a continuously distributed and sampled subspecies (*V. u. tasmaniensis*), and (c) assess the genetic provenance of the Maria Island population with respect to conservation genetic resources of *V. u. ursinus*. Discovery of genetically distinct populations across the wombat range will assist in determining spatial units that warrant independent management and support ongoing conservation planning for this Australian marsupial.

## 2 METHODS

### 2.1 Sampling locations and tissue collection

A total of 234 bare‐nosed wombat tissue samples was collected during 1999−2000 and 2014−2017, from the Australian mainland (*V. u. hirsutus*; *n* = 84), Bass Strait islands (Flinders Island; *V. u. ursinus*.; *n* = 10), Tasmania (*V. u. tasmaniensis*, *n* = 131) and Maria Island (subspecies uncertain; *n* = 9; Figure 1). Tissue samples were collected postmortem (road‐killed) or by live capture (via mesh nets or cage traps). Tissue was collected from the ear (central pinna) using a sterile 3‐mm biopsy punch (Kai Medical) and stored in 70% ethanol at −20°C until DNA extraction.

### 2.2 SNP discovery and filtering

High‐molecular‐weight DNA samples (*n* = 176), representative of the bare‐nosed wombat distribution, were sent to Diversity Arrays Technology Pty Ltd (DArT), Canberra, Australia, for DArTseq analysis. DArTseq utilizes complexity reduction (restriction enzymes *Pst*I and compliment, retained by DArT) and next‐generation sequencing methodologies to produce genome‐wide SNPs (Kilian et al., 2012; Sansaloni et al., 2011). A total of 28,081 SNPs were identified for *V. ursinus*. SNPs were filtered using the following exclusion criteria: reproducibility (<95%), missing data per locus (>20%), missing data per individual (>10%), secondaries (if multiple SNPs fall on the same sequence, removed the SNP with the lower read count average), minor allele frequencies (≤0.05), mean read depth per sample (<8) and heterozygosity (>0.5). Outlier SNPs identified according to both pcadapt (Luu, Bazin, & Blum, 2017) and sNMF (Frichot & François, 2015) were removed. Deviation from Hardy–Weinberg equilibrium (HWE) was assessed for three sampling regions in *Genepop* (Rousset, 2008): Tasmania, Maria and Flinders islands, and one mainland location (central Victoria). SNPs that were out of HWE in two or more of these sampling regions were removed from the data set (*n* = 372). This approach was taken to reduce the risk of mis‐identifying SNPs as out of HWE that are truly reflective of genetic structure (see Section 2.3 for comparative analyses performed including these SNPs). Filtering resulted in a total of 9,064 SNPs for 162 individuals (mainland, *n* = 76; Flinders, *n* = 6; Tasmania, *n* = 74; Maria Island, *n* = 6; Supporting information Data S1 and S2).

### 2.3 Diversity estimates and population structure

Heterozygosity, allelic richness and *F*_ST_ were estimated using the R packages *diveRsity* (Keenan, McGinnity, Cross, Crozier, & Prodöhl, 2013) and *strataG*(Archer, Adams, & Schneiders, 2017). Population structure was explored using a combination of multivariate and Bayesian methodologies. We focused on understanding structure at two different geographic scales: (a) among the three bare‐nosed wombat subspecies and (b) within the Tasmanian subspecies only, to reveal fine‐scale structure across a continuous sampling range. In each case, structure was assessed visually using principal component analysis (PCA, package adegenet V2.0.1; Jombart, 2008) and Bayesian cluster analysis (fastSTRUCTURE; Raj, Stephens, & Pritchard, 2014). All fastSTRUCTURE runs used a simple prior with cross‐validation (cv = 10) and explored *K* = 1−10 clusters. The optimal *K* range was determined using fastSTRUCTURE algorithms. PCA and fastSTRUCTURE were also performed including SNPs that violated our HWE filtering criterion for comparative purposes (Supporting information Data S3).

No additional structure analyses (beyond PCA and fastSTRUCTURE) were performed for the mainland region given the discrete spatial sample distribution and potential for false inference of genetic breaks if isolation by distance operates (Bradburd et al., 2018; Serre & Pääbo, 2004). However, further estimates of genetic diversity and differentiation were performed for the discrete populations located across the mainland (Supporting information Data S4). In Tasmania, where sampling was more continuous, a spatial principal component analysis (sPCA, package *adespatial*, Dray et al., 2018) was performed. sPCA incorporates both genetic variation and spatial autocorrelation (spatial weighting matrices) to explain observed patterns (Jombart, Devillard, Dufour, & Pontier, 2008). A Gabriel's graph was employed as the connection network, and sPCA scores were visually represented using the R package *ade4* (Dray & Dufour, 2007).

To complement the sPCA, we investigated isolation by distance in Tasmania by employing a redundancy analysis (RDA) following the methodology of Meirmans (2015). The RDA was performed as an individual—rather than population—based analysis, whereby the dependent variable was the allele count per locus per individual, and the independent variable was a set of spatial polynomials derived from geographic coordinates. It is important to note that potential landscape inhibitors to movement (e.g., lakes and rivers) are not considered by this approach. The RDA was performed in R using the package *VEGAN*(Oksanen et al., 2018).

## 3 RESULTS

### 3.1 Diversity estimates

Diversity estimates are described in Table 1. Eastern mainland locations (Victoria and New South Wales sites) had the highest allelic richness and observed heterozygosity (Ar = 1.56‒1.60, *H*_o_ = 0.19‒0.21), followed by Tasmania (Ar = 1.52, *H*_o_ = 0.18), and Maria and Flinders islands (Ar = 1.35‒1.39, *H*_o_ = 0.15‒0.16). The western mainland (South Australian location) had the lowest genetic diversity (Ar = 1.29, *H*_o_ = 0.11).

**Table 1:** Summary statistics for genome‐wide SNP loci (*n* = 9,064). See Figure 2a for locations

Region	*N*	*N*_I_	Ar	*H*_o_	*H*_e_
South Australia (SA)	5	4.74	1.29	0.11	0.14
Central Victoria (cVIC)	34	33.28	1.60	0.21	0.24
Eastern Victoria (eVIC)	15	14.59	1.57	0.20	0.23
New South Wales (NSW)	22	21.34	1.56	0.19	0.23
All Mainland	76	73.96	1.76	0.19	0.25
Flinders Is (FI)	6	5.85	1.39	0.15	0.16
Maria Is (MI)	6	5.80	1.35	0.16	0.15
Flinders and Maria Islands	12	11.65	1.46	0.15	0.17
Tasmania (TAS)	74	71.87	1.52	0.18	0.21

Note

Number of individuals (*N*), mean number of individuals typed per locus (*N*_I_), mean allelic richness (Ar), mean observed heterozygosity (*H*_o_) and mean expected heterozygosity (*H*_e_).

### 3.2 Population structure

Pairwise fixation indices estimated among regions (pooled locations: mainland, Flinders and Maria islands, and Tasmania) ranged from 0.24 to 0.33 (Table 2), and all were significant (*p* ≤ 0.01) after correction for false discovery rates. The mainland was less differentiated from Flinders and Maria islands (*F*_ST_ = 0.24) than it was from Tasmania (*F*_ST_ = 0.32), and Tasmania was most differentiated from the Flinders and Maria islands (*F*_ST_ = 0.33). Within the mainland, central Victoria (cVIC), eastern Victoria (eVIC) and New South Wales (NSW) had lower population differentiation (*F*_ST_ = 0.07–0.11), but experienced higher differentiation from South Australia (SA; *F*_ST_ = 0.21–0.25). Differentiation was also assessed at the population level within mainland groupings (Supporting information Data S4). Flinders Island and Maria Island (MI) showed very little genetic differentiation (*F*_ST_ = 0.05), and their differentiation from other populations was similar (Table 2).

**Table 2:** Pairwise *F*_ST_ among sampling regions derived from SNPs (left) and corresponding *p*‐values (right; corrected using the Benjamini–Hochberg method). All comparisons were significant (≤0.01). Among‐region comparisons are in bold. See Figure 2a for locations

	Mainland				Islands		Tasmania	All Mainland	Flinders and Maria
	South Australia	Central Victoria	Eastern Victoria	New South Wales	Flinders Is.	Maria Is.			
Mainland									
South Australia (SA)	–	0.009	0.009	0.009	0.010	0.010	0.009	–	–
Central Victoria (cVIC)	0.212	–	0.009	0.009	0.009	0.009	0.009	–	–
Eastern Victoria (eVIC)	0.229	0.074	–	0.009	0.009	0.009	0.009	–	–
New South Wales (NSW)	0.248	0.107	0.079	–	0.009	0.009	0.009	–	–
Islands									
Flinders Is. (FI)	0.426	0.264	0.267	0.276	–	0.010	0.009	–	–
Maria Is. (MI)	0.458	0.284	0.291	0.298	0.047	–	0.009	–	–
Tasmania (TAS)	0.416	0.351	0.352	0.361	0.317	0.334	–	**0.009**	**0.009**
All Mainland	–	–	–	–	–	–	**0.320**	–	**0.009**
Flinders and Maria	–	–	–	–	–	–	**0.325**	**0.241**	–

**Figure 2:**
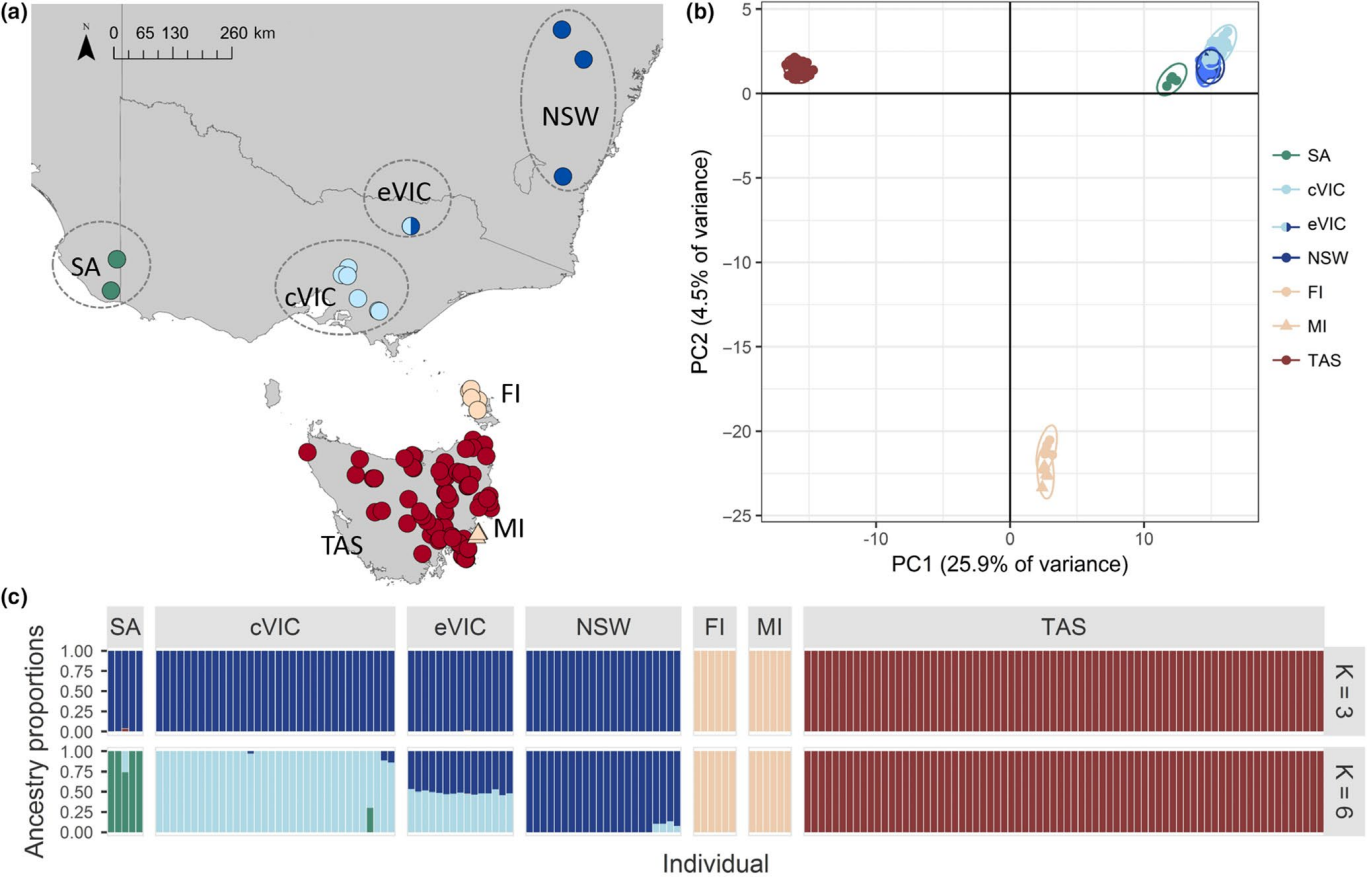
Genetic structuring of bare‐nosed wombats. Sample geographic locations (a) with colours corresponding to the results from PCA (b) and fastSTRUCTURE (c). Sampling location codes are as follows: South Australia (SA), central Victoria (cVIC), eastern Victoria (eVIC), New South Wales (NSW), Tasmania (TAS), Flinders Island (FI) and Maria Island (MI). PCA plot includes a 99% confidence ellipse for each location

**Figure 3:**
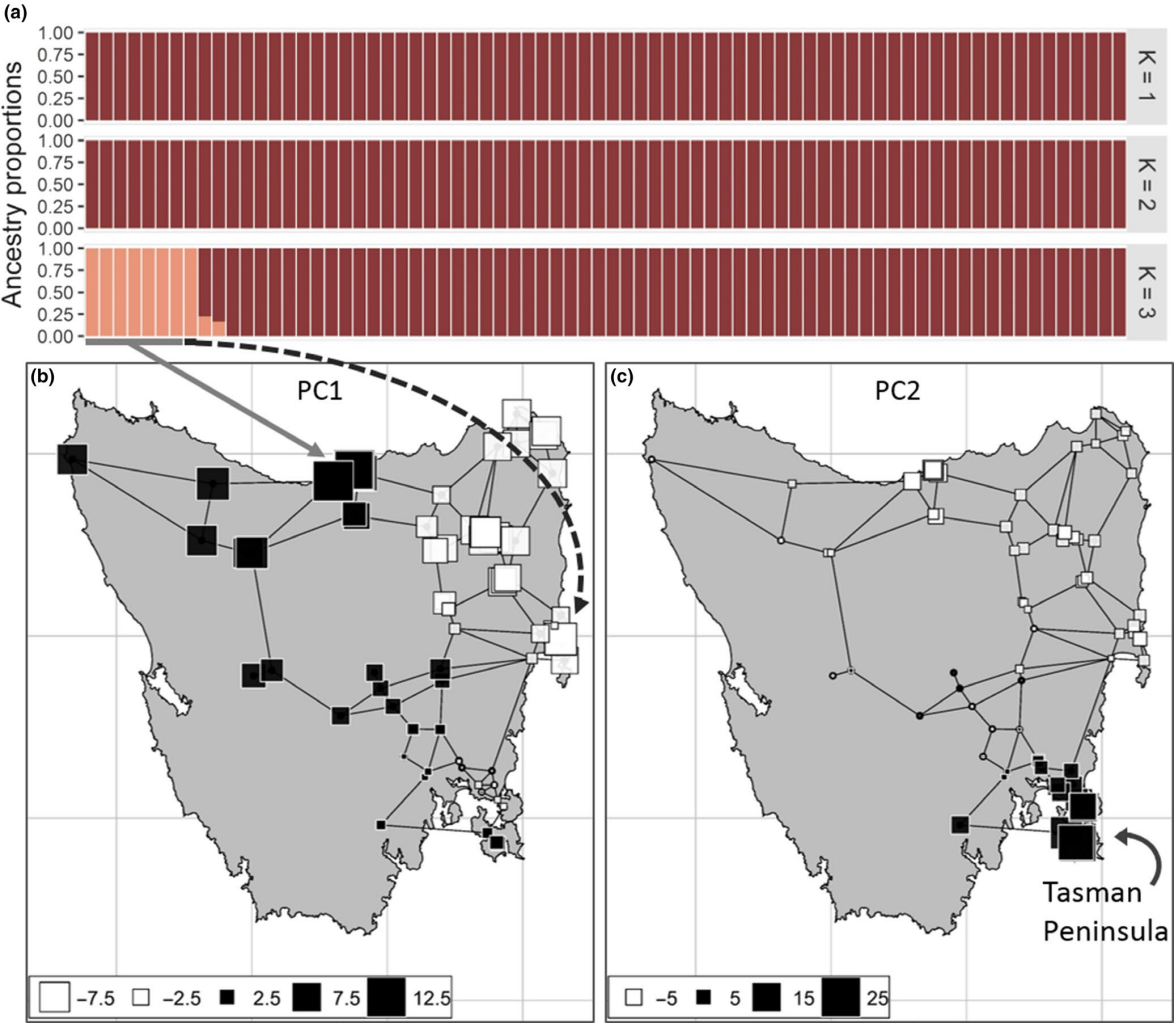
fastSTRUCTURE (a) and spatial principal component (sPCA) (b and c) results for Tasmanian individuals. Spatial mapping of the principal components 1 (b) and 2 (c) of the sPCA visually represents genetic differentiation proportional to difference in square size and shade. Arrows designate where the eight individuals assigned to the separate cluster (>99% ancestry proportion; a) are geographically located

Principal component analysis revealed three nonoverlapping clusters, with PC1 and PC2 explaining 25.9% and 4.5% of the variance, respectively. The groupings were as follows: (a) all Tasmanian individuals, (b) Maria Island and Flinders Island individuals, and (c) all mainland individuals. The fastSTRUCTURE analysis produced results consistent with the PCA when *K* was set to 3 (Figure 2, Supporting information Data S5), with assignment plots corresponding to the groups from the PCA. Additional structure was assessed for *K* = 5−6, the *K* range suggested by fastSTRUCTURE. *K* = 5−6 consistently grouped all Tasmanian samples together and Maria and Flinders Islands samples together, with further sub‐structuring suggested among mainland locations (Figure 2, Supporting information Data S6). PCAs and fastSTRUCTURE were also performed independently for the mainland, Flinders and Maria Islands, and Tasmania (Figure 3a; Supporting information Data S7‐S9), but no additional structure was only observed in Tasmania.

fastSTRUCTURE suggested a *K* range from 1 to 3 for Tasmania (Figure 3a), though most individuals were assigned to the same cluster. fastSTRUCTURE ancestry proportions clustered eight individuals—predominately from north‐central Tasmania—into a separate cluster when *K* = 3 (each having >99% of their ancestry assigned to this cluster). One of the eight individuals in the separate cluster was spatially discordant (from the east). sPCA revealed significant global structure in Tasmania (*p* < 0.01), but no local structure (*p* = 0.95). Individuals were genetically similar to those sampled adjacently, with the exception of east–west comparisons across north‐central Tasmania (PC1 34.8%; Figure 3b). Principal component 2 (PC2 30.7%; Figure 3c) showed differentiation of south‐eastern Tasmania (Tasman Peninsula and surroundings). RDA revealed significant correlation between genetic variation and geographic coordinates of samples (17.6% of the genetic variation explained by geographic coordinates, *p* = 0.001).

## 4 DISCUSSION

### 4.1 Genetic differentiation among *V. ursinus* subspecies

Designations of *V. ursinus* subspecies originated in the mid‐1800s and early 1900s when differences in body and skull size were observed in the geographically separated groups. The mainland subspecies was described as the largest, and Flinders Island *V. ursinus* were the smallest (Tate, 1951). While body size is often a distinguishable feature between island populations and their mainland conspecifics (Lomolino, 1985), observed differences between groups do not necessarily denote genetic divergence (Thomson et al., 2018). Here, our genome‐wide SNP analyses identified three genetic groups of *V. ursinus* that correspond to the presently recognized subspecies: mainland, *V. u. hirsutus*; Bass Strait, *V. u. ursinus*; and Tasmania, *V. u. tasmaniensis*.

Continental islands of Australia have been geographically separated from the mainland by sea‐level rise for ~6–17 kyr (Coller, 2007), and genetic differentiation among island and mainland populations has been observed in several instances (e.g., Kangaroo Island, Morris et al., 2018). Several species exhibit significant genetic divergence across Bass Strait: Bennett's wallaby, *Macropus rufogriseus* (Le Page, Livermore, Cooper, & Taylor, 2001); spotted‐tailed quoll, *Dasyurus maculatus* (Firestone et al., 1999); and platypus, *Ornithorhynchus anatinus*(Furlan et al., 2010; Gongora et al., 2012). Lowered genetic diversity has also been observed when compared to mainland lineages (platypus, *O. anatinus*; Furlan et al., 2012), which is a pattern commonly observed in island populations (Frankham, 1997). However, marine barriers have not influenced genetic structure for all species, such as the grey kangaroo, *Macropus giganteus* (Zenger, Eldridge, & Cooper, 2003), wedge‐tailed eagle, *Aquila audax*(Burridge et al., 2013), and white‐bellied sea eagle, *Haliaeetus leucogaster*(Shephard et al., 2005). Genetic structure (or lack‐there‐of) during comparisons of mainland and continental island populations may be influenced by several factors, including species dispersal capability and the environmental suitability of the land bridge.

It is evident that marine barriers have impacted the genetic structure of bare‐nosed wombats over and above that observed in their absence (e.g., divergence observed among subspecies compared to within). However, the genetic divergence of these populations does not immediately align with our current understanding of historical marine isolation. Specifically, the reconstruction of the southern coastline of Australia suggests that the flooding of the Bassian Plain separated the mainland from both Tasmania and Flinders Island first, while a land bridge still connected Tasmania and Flinders Island for an additional ~5–7 kyr (Coller, 2007; Lambeck & Chappell, 2001). However, mainland and Flinders Island subspecies exhibit less genetic distinction from each other than when compared to Tasmania. Two plausible explanations exist for these patterns. First, it is possible that gene flow across the Bassian Plain was influenced by factors other than sea level, and that despite being physically connected, geneflow was not achieved between Tasmania and Flinders Island following their isolation from the mainland. Second, *F*_ST_ is influenced by both population size and gene flow (Meirmans & Hedrick, 2011), and thus, a combination of our sample sizes and the population sizes may have influenced the genetic divergence observed. Therefore, estimates of divergence time are required to assess whether marine barriers initiated or reinforced the isolation of these populations (e.g., Burridge et al., 2013), and should be pursued in future analyses.

### 4.2 Genetic structure within subspecies

Within Tasmania, where sampling was more continuous, there was evidence for isolation by distance. While bare‐nosed wombats are capable of dispersal across varied landscapes (as their distribution suggests), they exhibit relatively small home ranges (on average 17.7 ha; Evans, 2008). Furthermore, dispersal is female‐biased in all wombat species (Banks et al., 2002; Johnson & Crossman, 1991; Walker, Taylor, & Sunnucks, 2008), and though the extent of these movements is not well understood, there is molecular‐ and tracking‐based evidence that suggests they are of short distances (100‒3,000 m). These short‐distance dispersal behaviours may provide some explanation for the isolation by distance observed within Tasmania. The exception to this pattern was observed in east–west comparisons in the north‐central region of Tasmania, where geographically close individuals were genetically dissimilar, in a manner akin to a “ring species” (Irwin, Irwin, & Price, 2001). This likely reflects long‐term barriers to gene flow present in this region, such as the Tamar River, with more recent (and likely weaker) impact from urbanization (the city of Launceston, the second largest city in Tasmania) and degraded landscapes (agricultural lands). Future research should investigate landscape features at finer scales to disentangle the potential contributors to this genetic break.

While most Tasmanian individuals were assigned to the same population cluster (*n* = 66, >90% ancestry assigned to the same cluster), it is worth noting that eight individuals were assigned (>99% ancestry) to a separate population cluster. Seven of these individuals were from the Tamar Valley region (north‐central Tasmania), specifically Narawntapu National Park and Greens Beach area. These locations are geographically close (<20 km) and well sampled in consecutive years due to research conducted in the area (Martin, Burridge, Ingram, Fraser, & Carver, 2018). Thus, this genetic cluster may reflect sampling of close relatives. The eighth individual assigned to this cluster was geographically distant and may reflect a translocation event resulting from wildlife rescue. Current wombat rehabilitation guidelines suggest a release site near the individual's capture location, but this is not always possible, and thus it is not uncommon that an individual is raised or rehabilitated and released in a different location. This individual was not distinguished in the sPCA results: the discrepancy between analyses may reflect a lack of spatial information incorporated into fastSTRUCTURE and reveals potential limitations in identifying migrant (or translocated) individuals using sPCA.

Though our mainland sampling was more spatially discrete, which places constraints on the interpretation of genetic structuring (Bradburd et al., 2018), we found high genetic differentiation within *V. u. hirsutus* specifically against the South Australian samples (SA). This longitudinal pattern of genetic differentiation is consistent with previous studies of *V. u. hirsutus*, using microsatellite loci (Banks et al., 2002). This may be reflective of the recent fragmentation across the western range of *V. u. hirsutus* (IUCN, 2016), as the eastern mainland is less differentiated over comparable spatial scales. Further, the SA population is likely smaller and thus more susceptible to genetic drift (Frankham, 1996). These patterns may also be observed in the fragmented range in southern Queensland and northern New South Wales; however, samples from these regions were absent from our analyses. Finer spatial sampling across the mainland is required to determine factors responsible for genetic structuring in this region.

### 4.3 *V. u. ursinus* on Maria Island

Bare‐nosed wombats have been subjected to considerable human interference across the Bass Strait islands, becoming extinct on King, Cape Barren, Flinders, Deal and Clarke islands. Given this history, *V. u. ursinus* was listed as Vulnerable in 2008 under the *Environment Protection and Biodiversity Conservation Act 1999* (Commonwealth EPBC). However, we reveal a second population of *V. u. ursinus*located on Maria Island. Following the translocation event of 1971, wombats on Maria Island were considered rare (Rounsevell et al., 1991). However, the present population is prolific and has experienced growth over the last decade (Ingram, 2015). The Maria Island population has two implications for the conservation of *V. u. ursinus*: (a) it represents security for the future of *V. u. ursinus* and (b) indicates the potential ease at which *V. u. ursinus* could be re‐introduced to Bass Strait islands.

We observe no genetic signature of multiple *V. ursinus* subspecies in the Maria Island population, suggesting either (a) *V. u. tasmaniensis* was present at the time of translocation but no genetic signature has been retained to present, or (b) this lineage was not present at the time of translocation. If wombats were already present at the time of the Flinders translocation event, their low abundance may have reflected inbreeding depression (Frankham, 2010), and the translocation may have constituted a genetic rescue event (Frankham, Handasyde, & Eldridge, 2016; Whiteley, Fitzpatrick, Funk, & Tallmon, 2015). Despite founding by only 21 individuals, genetic diversity in the Maria Island population was comparable to that of Flinders Island. This is supported by similar estimates of allelic richness and the low pairwise fixation index. Therefore, this translocation event may have captured most of the genetic variation on Flinders Island. However, it is possible that Flinders Island has experienced a loss in diversity since the translocation event, resulting in similar diversity estimates to Maria Island, which are low compared to Tasmania and the mainland. Furthermore, the Flinders–Maria fixation index is significantly greater than zero and may indicate important genetic differentiation, or in this case, may be reflective of a founder effect or genetic drift (Weeks, Stoklosa, & Hoffmann, 2016). The lowered genetic diversity (allelic richness) observed in both Maria and Flinders islands populations, and to a lesser extent in Tasmania, is typical of island populations (Frankham, 1997), but may require management action if low fitness is observed in the future (i.e., genetic rescue; Frankham, 2015; Whiteley et al., 2015).

### 4.4 Applied evolutionary management

There is ongoing debate regarding the genetic identification of intraspecific units warranting independent conservation (Coates, Byrne, & Moritz, 2018). Given the identification of three genetically and phenotypically distinct wombat lineages across geographically (and reproductively) isolated regions, it may be appealing to consider the subspecies separately for management purposes, as legislation often considers subspecies as separate entities for conservation (Coates et al., 2018). Significant genetic divergence was also observed among recently fragmented mainland wombat populations. However, neutral genetic divergence among populations may not necessarily reflect adaptive differences (Coates et al., 2018; Crandall, Bininda‐Emonds, Mace, & Wayne, 2000; Ralls et al., 2018) and could instead reflect the action of genetic drift during population declines, concomitantly reducing genetic diversity. Under such circumstances, management to maintain genetic distinctiveness of populations could increase their extinction risk if they suffer from low fitness, potentially reflecting inbreeding depression or genetic load (Hedrick & Fredrickson, 2010; Ralls et al., 2018; Weeks et al., 2016). Research on bare‐nosed wombats to assess fitness and adaptive distinction has been insufficient, although dramatic population declines have been observed in some areas (e.g., in response to novel pathogens; Martin et al., 2018). As additional resources become available (i.e., the annotation of the wombat genome), questions regarding adaptive distinction can also be investigated more thoroughly (Pardo‐Diaz, Salazar, & Jiggins, 2015). Regardless, if fitness is low, there are potential benefits through the incorporation of genetic variation from other populations (“genetic rescue”; Frankham, 2015; Ralls et al., 2018). However, controlled crosses need first be conducted to assess potential fitness benefits, and the risk of outbreeding depression (although these appear overstated, generally; Frankham et al., 2011). The Bass Strait islands previously harbouring *V. u. ursinus* provide an ideal opportunity to both establish additional insurance populations of pure *V. u. ursinus*, and also to test the potential fitness benefits of crosses within and between subspecies, if indeed natural populations are ascertained to be threatened by low fitness.

## CONFLICT OF INTEREST

The authors declare no competing interests.

## ANIMAL ETHICS

Tissue collection was approved by the Animal Ethics Committee at the University of Tasmania (A14670), the Department of Primary Industries, Parks, Water and Environment (FA15003, FA15122), Monash University (BSci2000/09), Melbourne University Veterinary Science Animal Experimentation Ethics Sub‐Committee (#98108) and Australian National University (A2016/08).

## DATA AVAILABILITY

Reference sequences and SNP genotypes are available at the Dryad Digital Repository: https://doi.org/10.5061/dryad.5t37q5f.
