# Supplementary material for: Isolation, marine transgression and translocation of the bare‐nosed wombat (Vombatus ursinus)
Source: Evol Appl. 2019 Mar 21;12(6):1114–23. doi: 10.1111/eva.12785 (PMC6597867; doi:10.1111/eva.12785)
Supplement: Supplementary file 1 [file EVA-12-1114-s001.docx]

Supplementary Materials

Population structure in the bare-nosed wombat

Data S1. Bare-nosed wombat sample location details (total of 162; sample size per location, N).

| State | N | Latitude | Longitude | Sample ID |
| --- | --- | --- | --- | --- |
| Flinders Is | 1 | -39.995 | 148.060 | F4 |
| Flinders Is | 1 | -39.821 | 147.883 | Reg1 |
| Flinders Is | 1 | -39.819 | 147.882 | Reg2 |
| Flinders Is | 1 | -39.778 | 147.916 | Reg3 |
| Flinders Is | 1 | -39.955 | 147.925 | Reg4 |
| Flinders Is | 1 | -40.195 | 148.047 | Reg6 |
| Maria Is | 2 | -42.581 | 148.066 | M1_ms |
| Maria Is | 3 | -42.661 | 148.024 | MX_ms |
| Maria Is | 1 | -42.593 | 148.052 | M9_ms |
| New South Wales | 18 | -35.571 | 149.739 | Vur_GS_X |
| New South Wales | 3 | -33.255 | 150.148 | Vur_JO_X |
| New South Wales | 1 | -32.667 | 149.717 | Vur_SB_004 |
| South Australia | 1 | -37.828 | 140.780 | Vur_SB_021 |
| South Australia | 4 | -37.211 | 140.898 | Vur_TC_X |
| Tasmania | 1 | -41.106 | 146.791 | 391 |
| Tasmania | 3 | -41.090 | 146.754 | 397 |
| Tasmania | 1 | -42.898 | 147.813 | Am11 |
| Tasmania | 1 | -41.810 | 147.409 | Am12 |
| Tasmania | 1 | -42.684 | 147.530 | Bek |
| Tasmania | 1 | -42.662 | 147.122 | BN01 |
| Tasmania | 1 | -41.818 | 147.422 | Cam1 |
| Tasmania | 1 | -41.343 | 146.775 | CB1 |
| Tasmania | 1 | -42.217 | 146.014 | CB12 |
| Tasmania | 1 | -41.476 | 145.632 | CB13 |
| Tasmania | 1 | -42.437 | 146.654 | CB15 |
| Tasmania | 1 | -42.189 | 146.148 | CB16 |
| Tasmania | 1 | -41.518 | 147.419 | CB17 |
| Tasmania | 1 | -41.531 | 147.355 | CB18_ms |
| Tasmania | 1 | -42.245 | 147.405 | CB2 |
| Tasmania | 1 | -43.081 | 147.739 | CB3 |
| Tasmania | 1 | -42.817 | 147.789 | CB4 |
| Tasmania | 1 | -42.514 | 147.391 | CB5 |
| Tasmania | 1 | -42.688 | 147.529 | CB6 |
| Tasmania | 1 | -42.741 | 147.772 | CB8 |
| Tasmania | 1 | -43.140 | 147.820 | CH004 |
| Tasmania | 1 | -43.134 | 147.807 | Ch006 |
| Tasmania | 1 | -42.857 | 147.697 | CR1 |
| Tasmania | 1 | -42.134 | 148.309 | FNP1 |
| Tasmania | 1 | -41.979 | 148.239 | FNP2 |
| Tasmania | 1 | -41.888 | 148.279 | FNP3_ms |
| Tasmania | 2 | -42.017 | 148.279 | FNPX |
| Tasmania | 1 | -41.544 | 145.974 | Grob1 |
| Tasmania | 1 | -41.543 | 146.001 | Grob7 |
| Tasmania | 1 | -42.818 | 147.672 | H2 |
| Tasmania | 1 | -42.941 | 147.862 | Jbog1 |
| Tasmania | 1 | -42.935 | 147.859 | Jbog2 |
| Tasmania | 1 | -41.226 | 147.400 | KS10 |
| Tasmania | 1 | -41.703 | 147.851 | KS2 |
| Tasmania | 1 | -41.470 | 147.819 | KS3 |
| Tasmania | 1 | -41.458 | 147.792 | KS4 |
| Tasmania | 1 | -41.419 | 147.647 | KS5 |
| Tasmania | 1 | -41.330 | 146.757 | KS7 |
| Tasmania | 1 | -43.037 | 146.953 | KS9 |
| Tasmania | 1 | -40.780 | 147.955 | MtW1 |
| Tasmania | 1 | -40.899 | 148.154 | MtW2 |
| Tasmania | 1 | -42.775 | 147.277 | Old B |
| Tasmania | 1 | -41.165 | 145.712 | RKW003 |
| Tasmania | 1 | -41.988 | 148.129 | SC1 |
| Tasmania | 1 | -42.512 | 147.190 | SC10 |
| Tasmania | 1 | -40.875 | 148.175 | SC13 |
| Tasmania | 1 | -40.942 | 147.953 | SC14 |
| Tasmania | 1 | -42.123 | 148.067 | SC15 |
| Tasmania | 1 | -40.960 | 147.814 | SC16 |
| Tasmania | 1 | -41.107 | 148.218 | SC17 |
| Tasmania | 1 | -42.386 | 147.041 | SC2 |
| Tasmania | 1 | -42.744 | 147.301 | SC5 |
| Tasmania | 1 | -42.181 | 147.390 | SC6 |
| Tasmania | 1 | -42.287 | 146.951 | SC7 |
| Tasmania | 1 | -41.029 | 144.675 | SC8 |
| Tasmania | 1 | -42.722 | 147.551 | Scabies_ms |
| Tasmania | 1 | -42.201 | 146.905 | TL05 |
| Tasmania | 1 | -41.474 | 147.711 | TL06 |
| Tasmania | 1 | -41.399 | 147.293 | TL09 |
| Tasmania | 1 | -41.430 | 147.725 | TL1 |
| Tasmania | 1 | -41.959 | 147.498 | TL12 |
| Tasmania | 1 | -41.477 | 147.946 | TL2 |
| Tasmania | 2 | -41.430 | 147.725 | TLX |
| Tasmania | 1 | -41.696 | 147.862 | TT1 |
| Tasmania | 1 | -41.681 | 147.889 | TT2 |
| Tasmania | 1 | -41.944 | 148.236 | TT3 |
| Tasmania | 3 | -41.150 | 146.599 | W00X |
| Tasmania | 1 | -41.854 | 147.452 | WOM |
| Victoria | 15 | -37.376 | 145.484 | Vur_LS_X |
| Victoria | 15 | -36.556 | 146.724 | Vur_MW_1 |
| Victoria | 15 | -37.986 | 145.675 | Vur_SB_006 |
| Victoria | 1 | -37.518 | 145.359 | Vur_SB_023 |
| Victoria | 1 | -37.538 | 145.468 | Vur_SB_024 |
| Victoria | 1 | -38.225 | 146.078 | WW01 |
| Victoria | 1 | -38.237 | 146.098 | WW02 |

Data S2. Number of single nucleotide polymorphisms and individuals retained after applying each filtering step for the bare-nosed wombat (*Vombatus ursinus*) DArT data set.

| **Exclusion criteria (filtering step)** | **Individual count** | **SNP Count** |
| --- | --- | --- |
| Raw SNP data set | 165 | 28,081 |
| 1. Reproducibility (<95%) | 165 | 27,475 |
| 1. Missing data per locus (>20%) | 165 | 22,619 |
| 1. Secondaries   (If two SNPs fall on the same fragment, the SNP with the lower read count average is removed) | 165 | 18,329 |
| 1. Missing data per individual (>10%)   (Loci that become monomorphic due to excluded individuals are also removed) | 162 | 18,311 |
| 1. Minor allele frequency (≤0.05) | 162 | 10,760 |
| 1. Read depth (coverage depth <8) | 162 | 9,816 |
| 1. Heterozygosity (>0.5) | 162 | 9,778 |
| 1. Outliers identified by both PCADAPT and SNMF (significance < 0.05) | 162 | 9,436 |
| PCADAPT identified 1,034 SNPs |  |  |
| sNMF identified 1,029 SNPs |  |  |
| 1. Hardy-Weinberg dis-equilibrium in ≥2 locations (GENEPOP) | 162 | 9,064 |

Data S3. To explore the impact of filtering SNPs based on Hardy-Weinberg Equilibrium criteria, fastSTRUCTURE analysis and PCAs were performed including the 372 SNPs. fastSTRUCTURE results were largely the same (A), with the exception of the most likely number of clusters for the mainland. Assignment plots were created for the all samples (B) and mainland only (C). PCA results for all samples (D) and mainland only (E) are also shown.

A. Results of fastSTRUCTURE for K=1−10 for different sampling regions.

| K | Sampling region | | | |
| --- | --- | --- | --- | --- |
|  | All | Mainland only | Tasmania only | Maria & Flinders |
| 1 | -0.903080 | -0.808204 | **-0.680214** | **-0.715486** |
| 2 | -0.757455 | -0.800275 | -0.680783 | -0.716102 |
| 3 | -0.742196 | **-0.796945** | -0.697503 | -0.716342 |
| 4 | -0.738415 | -0.803683 | -0.696174 | -0.716474 |
| 5 | -0.738475 | -0.812736 | -0.712313 | -0.786648 |
| 6 | **-0.736798** | -0.819058 | -0.681264 | -0.716619 |
| 7 | -0.736846 | -0.812829 | -0.696735 | -0.716665 |
| 8 | -0.736886 | -0.811328 | -0.681340 | -0.716701 |
| 9 | -0.738654 | -0.808638 | -0.681367 | -0.716731 |
| 10 | -0.736942 | -0.812960 | -0.681390 | -0.716756 |
| K based on model complexity | 6 | 3 | 1 | 1 |
| K based on model components | 5 | 5 | 1 | 1 |


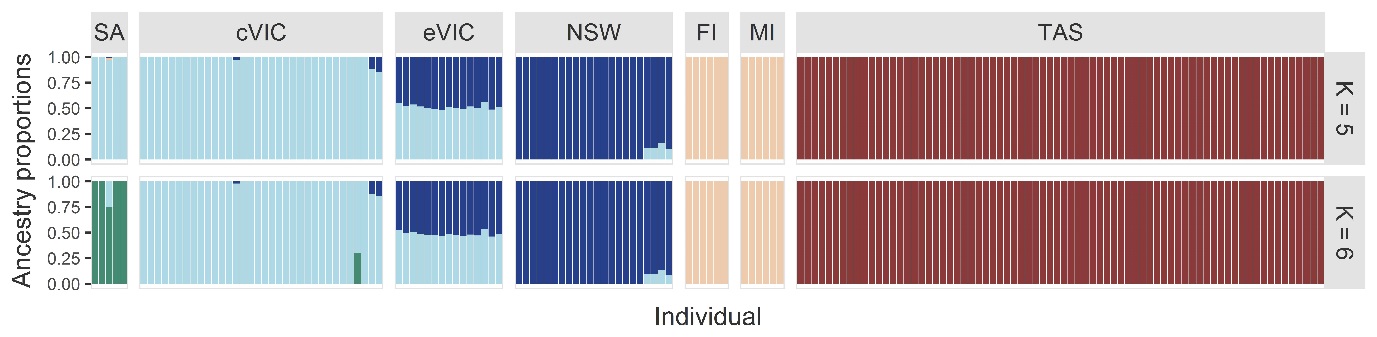


B


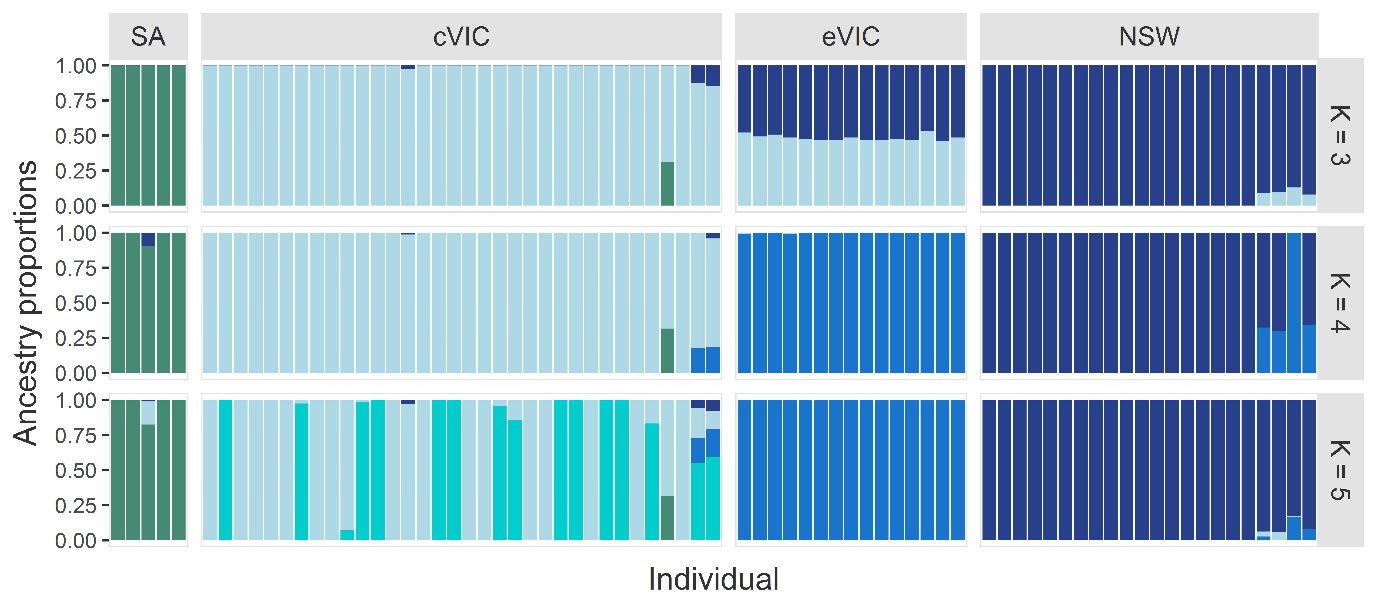


C


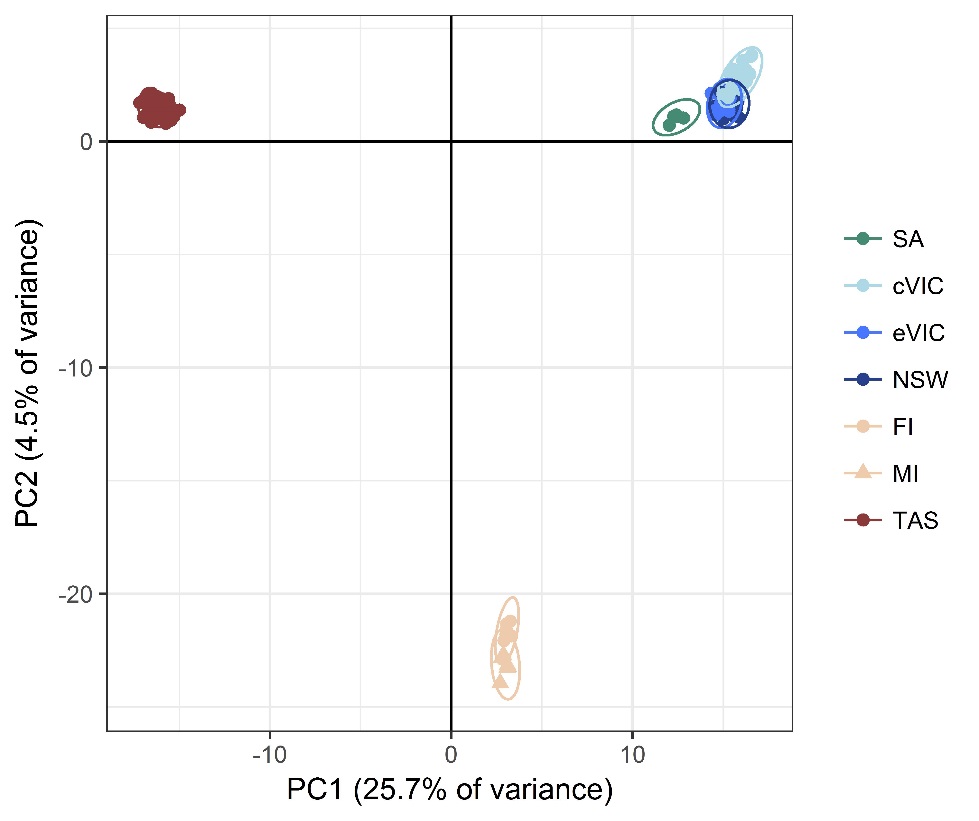


D


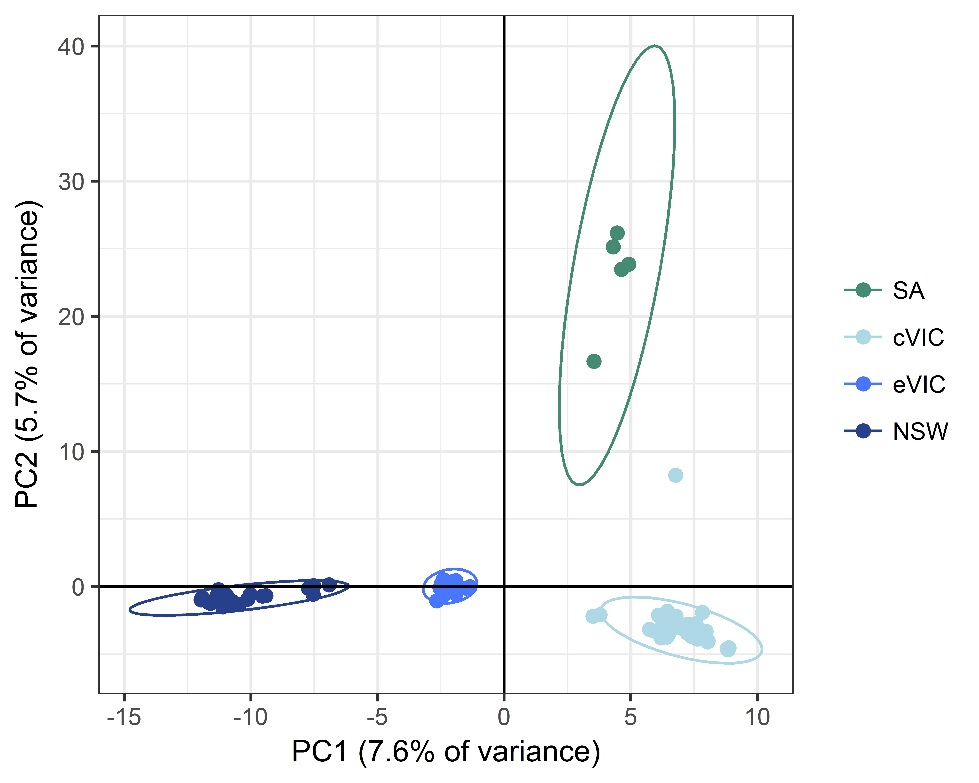


E

Data S4. Estimates of diversity and differentiation for mainland populations. ‘Populations’ were sites with ≥3 individuals sampled in the same location (n=6, labels upper right; A). Allelic diversity (B) and pairwise F_ST_ (C) were estimated for each population using the same methods described in the main text. An hierarchical analysis of molecular variance [AMOVA, package *poppr* (Kamvar et al. 2015)] was performed across population and regional (SA, cVIC, eVIC, and NSW; black dotted circles) levels (D).


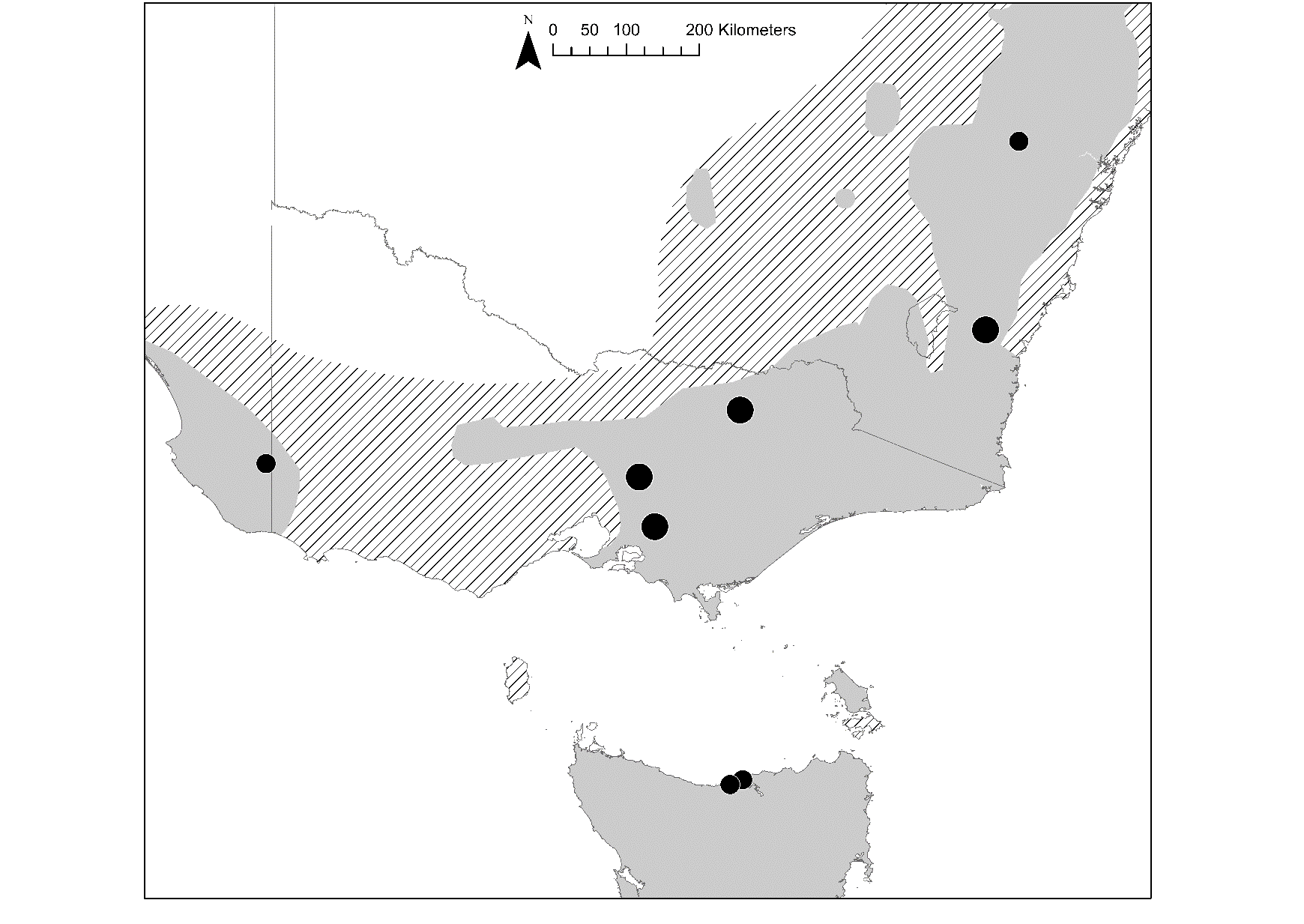


A

**SA**

**cVIC1**

**eVIC**

**cVIC2**

**NSW1**

**NSW2**

3-5

15-18

Sample size

Distribution

Current

Historical


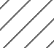

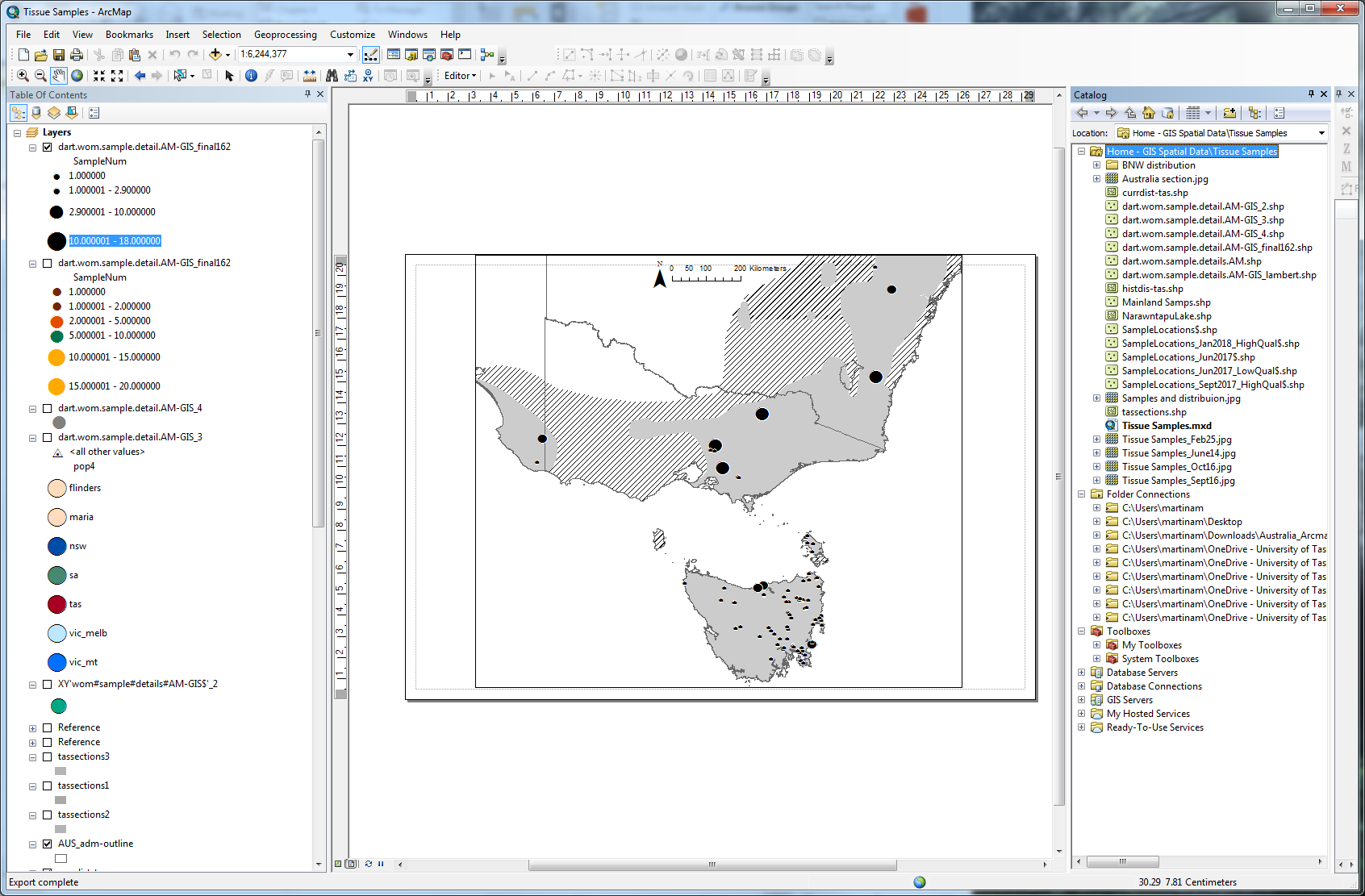

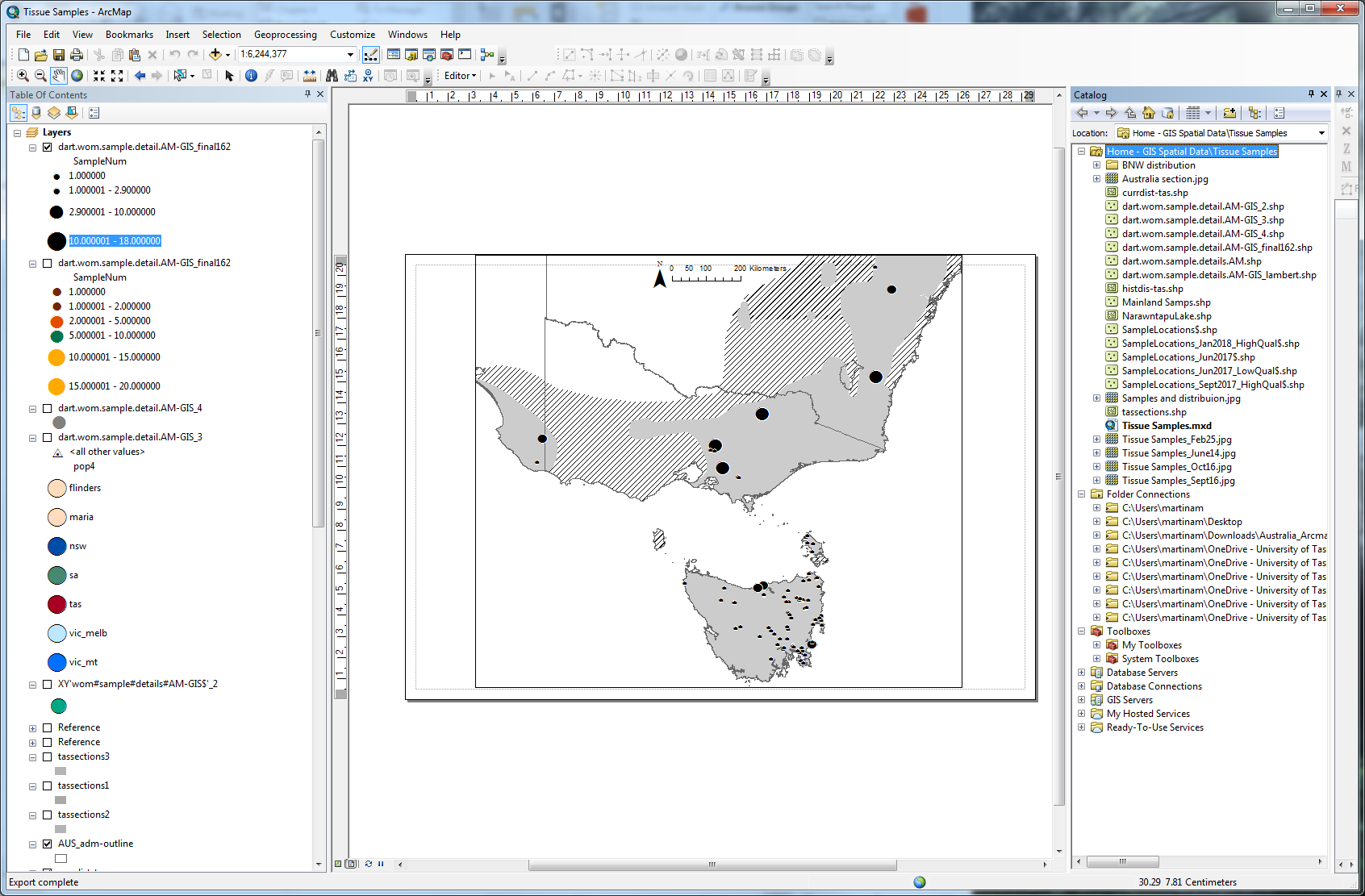


B. Summary statistics for genome-wide SNP loci (n=9064) for the six mainland populations.

| Region | N | N_I_ | Ar | H_o_ | | H_e_ |
| --- | --- | --- | --- | --- | --- | --- |
| South Australia (SA) | 4 | 3.81 | 1.24 | | - | 0.14 |
| Central Victoria 1 (cVIC1) | 15 | 14.67 | 1.48 | | 0.21 | 0.23 |
| Central Victoria 2 (cVIC2) | 15 | 14.59 | 1.48 | | 0.20 | 0.23 |
| Eastern Victoria (eVIC) | 15 | 14.69 | 1.47 | | 0.20 | 0.23 |
| New South Wales 1 (NSW1) | 18 | 17.46 | 1.46 | | 0.19 | 0.22 |
| New South Wales 2 (NSW2) | 3 | 2.91 | 1.35 | - | | 0.18 |

*Number of individuals (N), mean number of individuals typed per locus (N_I_), mean allelic richness (Ar), mean observed heterozygosity (H_o_), and mean expected heterozygosity (H_e_)*

C. Pairwise F_ST_ among sampling regions derived from SNPs (left) and corresponding *P*-values (right; corrected using the Benjamini-Hochberg method).

|  | SA | cVIC1 | cVIC2 | eVIC | NSW1 | NSW2 |
| --- | --- | --- | --- | --- | --- | --- |
| SA | - | 0.028 | 0.032 | 0.035 | 0.056 | 0.667 |
| cVIC1 | 0.214 |  | 0.032 | 0.011 | 0.011 | 0.545 |
| cVIC2 | 0.218 | 0.017 |  | 0.011 | 0.011 | 0.270 |
| eVIC | 0.212 | 0.078 | 0.083 |  | 0.011 | 0.667 |
| NSW1 | 0.247 | 0.121 | 0.127 | 0.089 | - | 1.00 |
| NSW2 | 0.299 | 0.130 | 0.137 | 0.098 | 0.084 | - |

D. Hierarchical analysis of molecular variance (AMOVA) results.

| Source of variation | Degrees of freedom | Sum of Squares | Sigma | Variation (%) | ϕ |
| --- | --- | --- | --- | --- | --- |
| Between regions | 3 | 16162.64 | 104.7978 | 8.74 | 0.087 |
| Between populations within regions | 2 | 3905.875 | 36.7944 | 3.07 | 0.033 |
| Between samples within populations | 64 | 77554.75 | 154.6858 | 12.90 | 0.146 |
| Within samples | 70 | 63169.5 | 902.4214 | 75.28 | 0.247 |
| Total | 139 | 160792.8 | 1198.699 | 100 | - |

Data S5. Results of fastSTRUCTURE for K=1−10 for different sampling regions when SNPs are filtered using HWE criteria. The K range was estimated by fastSTRUCTURE. Lowest marginal likelihood values are in bold.

| K | Sampling region | | | |
| --- | --- | --- | --- | --- |
|  | All | Mainland only | Tasmania only | Maria & Flinders |
| 1 | -0.896200 | -0.803221 | **-0.672007** | **-0.708640** |
| 2 | -0.750563 | -0.795673 | -0.672597 | -0.709274 |
| 3 | -0.735609 | **-0.792636** | -0.688460 | -0.709521 |
| 4 | -0.731975 | -0.799044 | -0.703129 | -0.709657 |
| 5 | -0.732066 | -0.803756 | -0.699714 | -0.709744 |
| 6 | **-0.730519** | -0.799238 | -0.703415 | -0.709806 |
| 7 | -0.730580 | -0.807129 | -0.691751 | -0.709854 |
| 8 | -0.730609 | -0.806565 | -0.691578 | -0.709891 |
| 9 | -0.730641 | -0.807264 | -0.682691 | -0.709922 |
| 10 | -0.730719 | -0.800579 | -0.673228 | -0.709948 |
| K based on model complexity | 6 | 3 | 1 | 1 |
| K based on model components | 5 | 4 | 3 | 1 |

Data S6. fastSTRUCTURE assignment plots for K=3−10 including all individuals. Lack of distinction between some results with different K values reflects individuals being allocated a very small ancestry to a cluster (e.g., 0.00002).


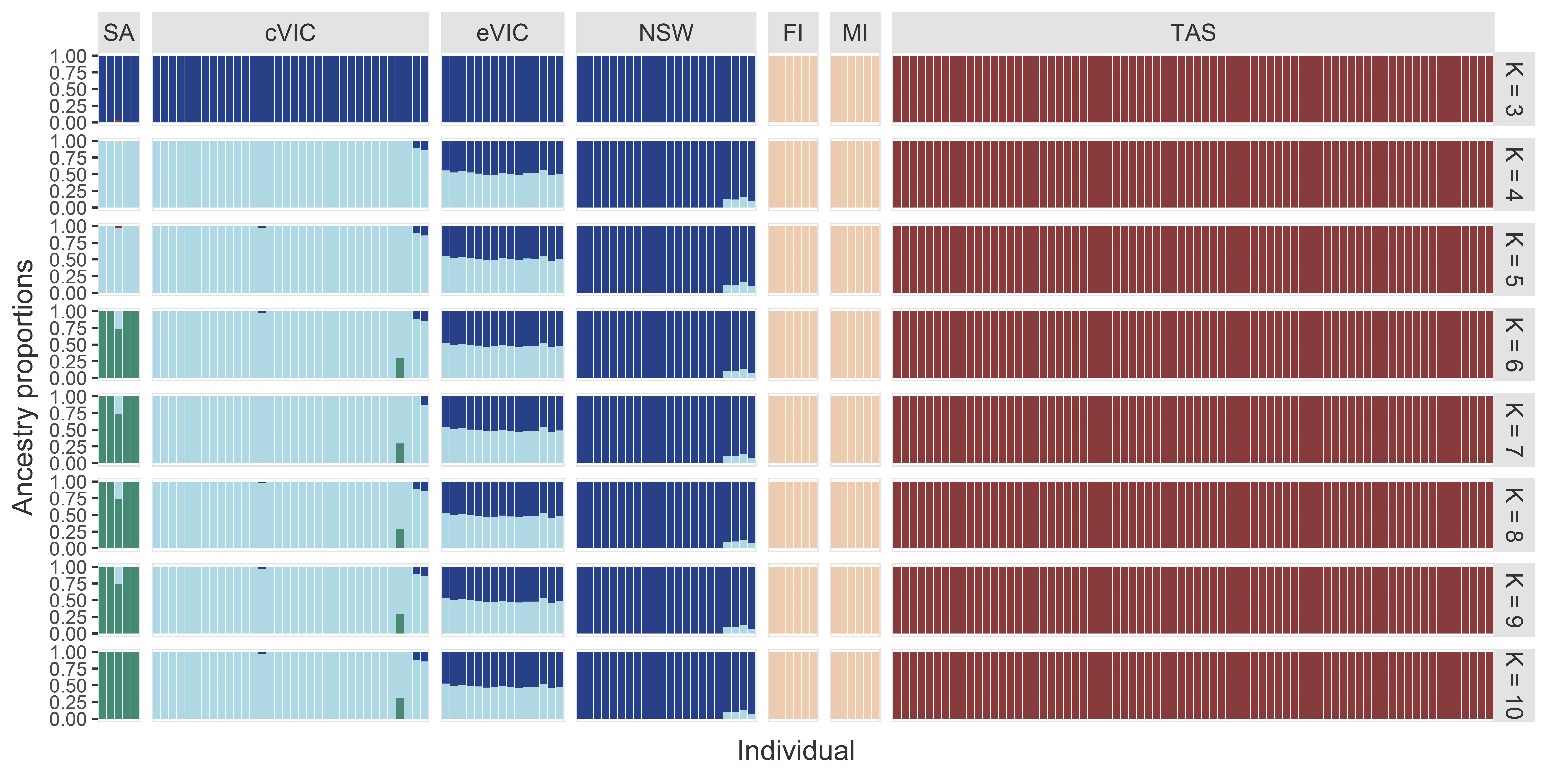


Data S7. The PCA (A) and fastSTRUCTURE (B) results for mainland individuals. Inset in the PCA graph is the corresponding eigenvalues, displayed as a bar plot. Each sampling region has a 99% confidence ellipse added.


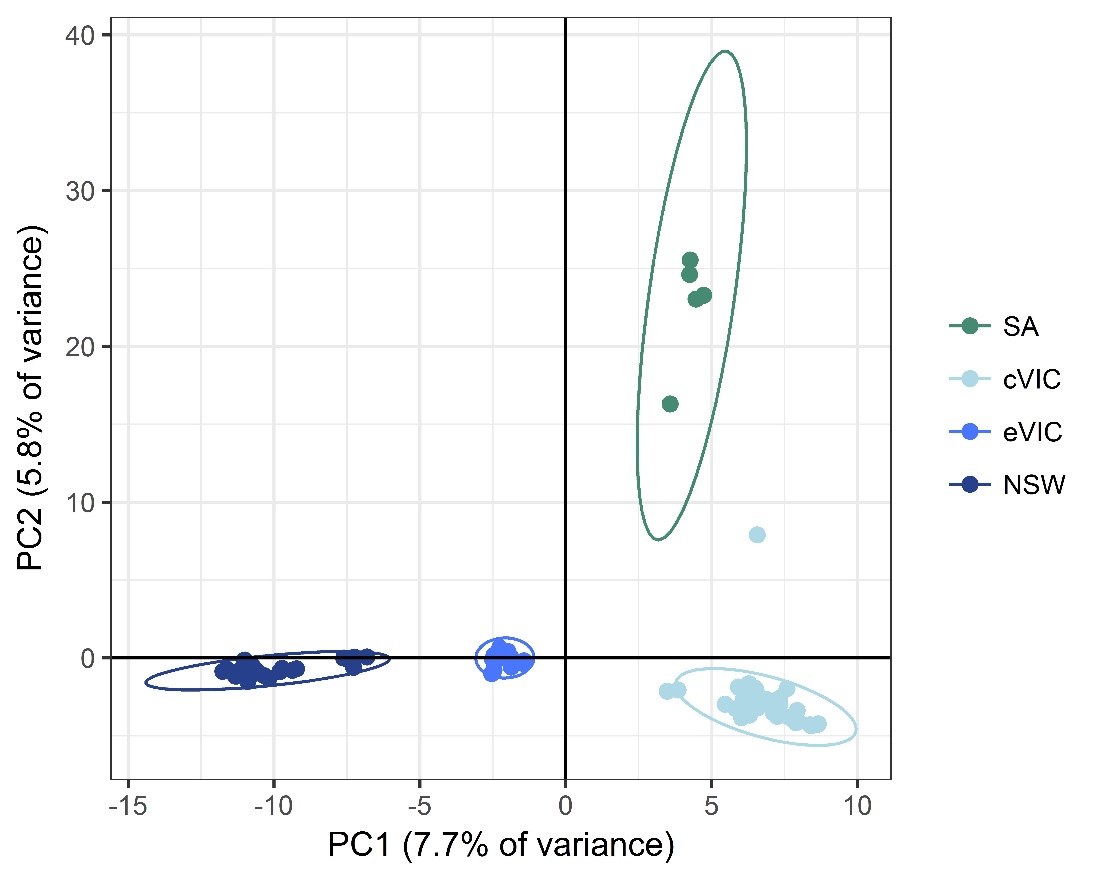


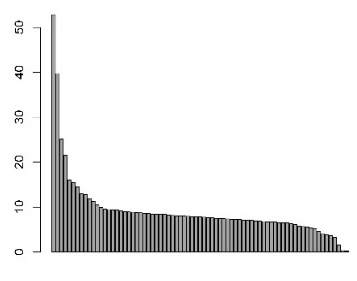


**A**


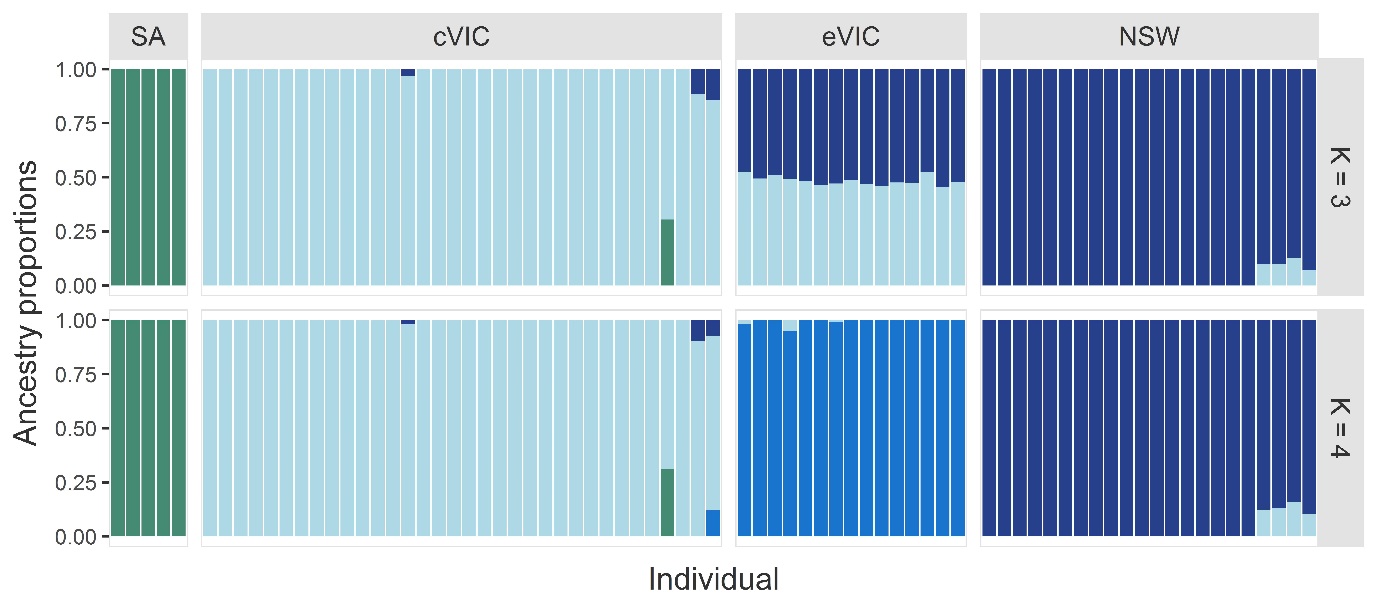


**B**

Data S8. The PCA (A) and fastSTRUCTURE (B) results for island (Flinders and Maria) individuals. Inset in the PCA graph is the corresponding eigenvalues, displayed as a bar plot. Each sampling region has a 99% confidence ellipse added.


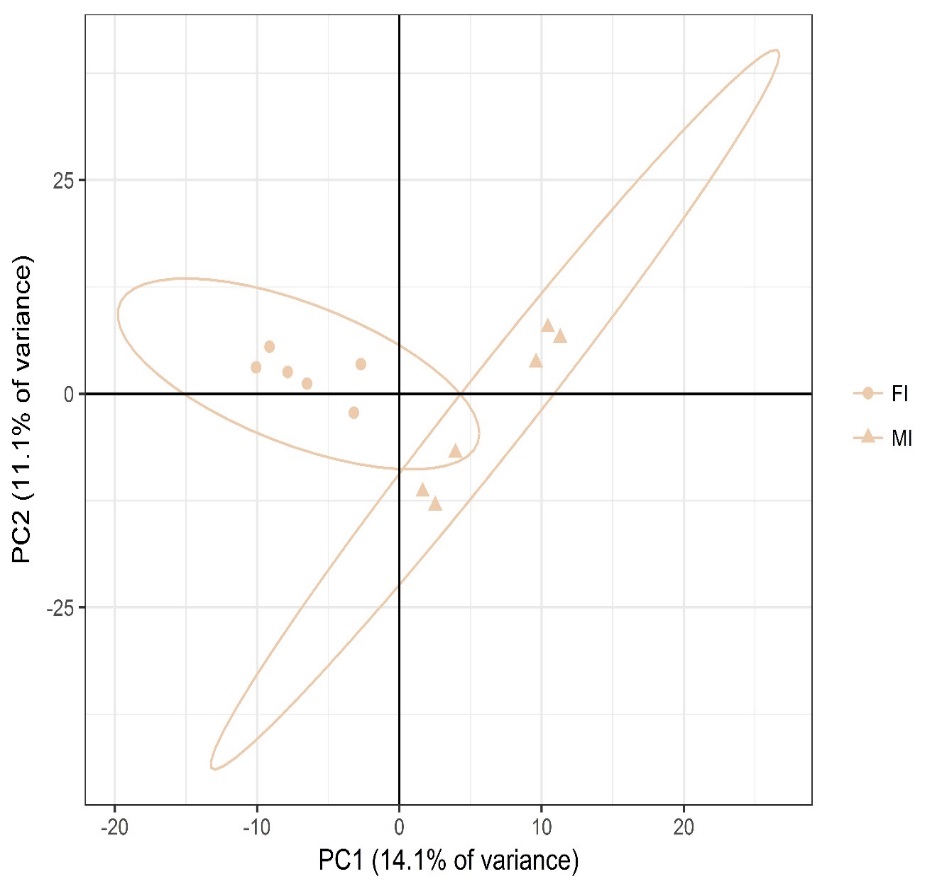

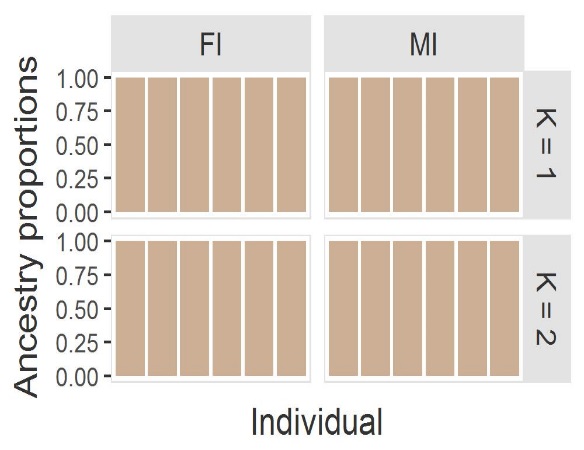


**B**

**A**


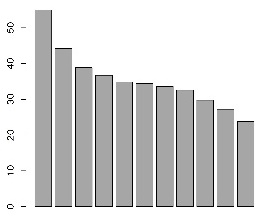


Data S9. The PCA results for Tasmanian individuals. Inset in the PCA graph is the corresponding eigenvalues, displayed as a bar plot. A 99% confidence ellipse is portrayed.


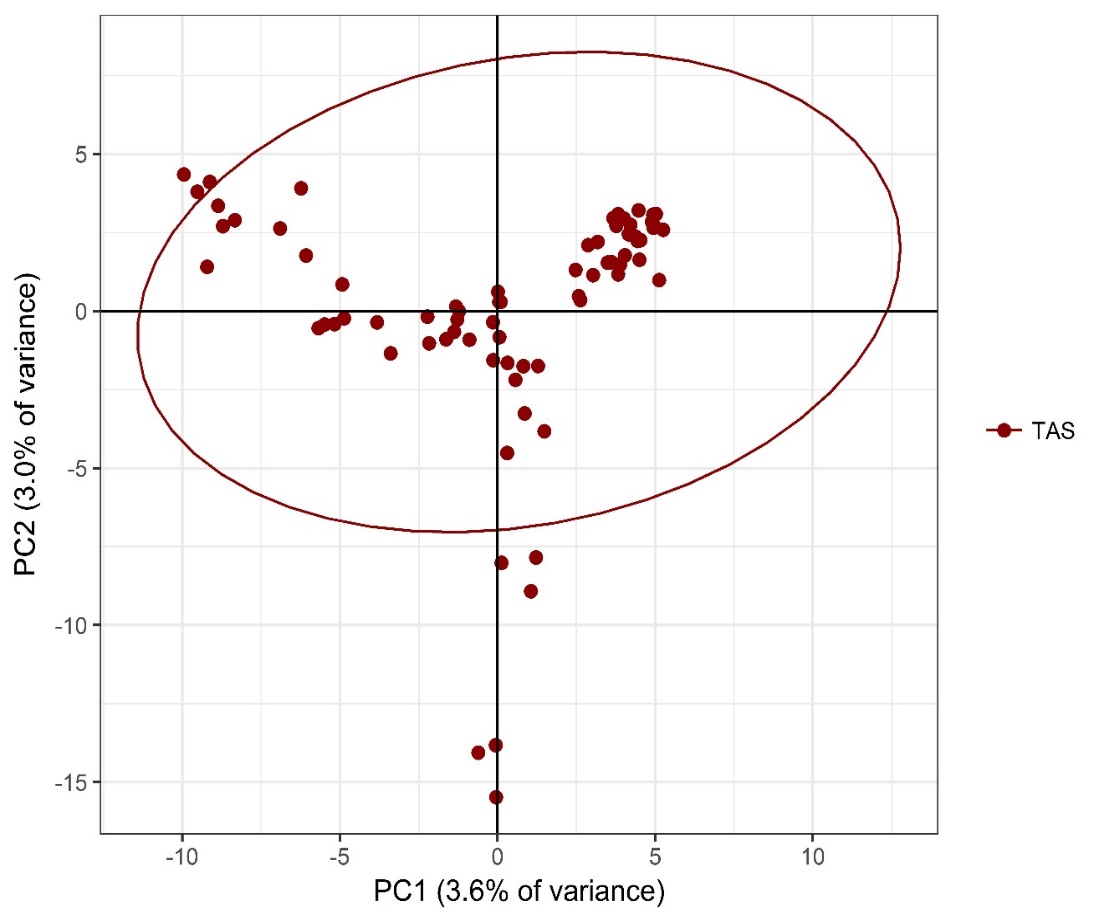

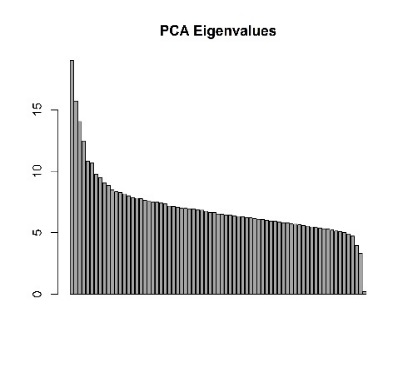


Literature cited

Kamvar, Z. N., J. C. Brooks, and N. J. Grünwald. 2015. Novel R tools for analysis of genome-wide population genetic data with emphasis on clonality. Frontiers in genetics **6**.
